# Return to knee-strenuous sport after anterior cruciate ligament reconstruction: a report from a rehabilitation outcome registry of patient characteristics

**DOI:** 10.1007/s00167-016-4280-1

**Published:** 2016-08-16

**Authors:** Eric Hamrin Senorski, Kristian Samuelsson, Christoffer Thomeé, Susanne Beischer, Jón Karlsson, Roland Thomeé

**Affiliations:** 1 Unit of Physiotherapy, Department of Health and Rehabilitation, Institution of Neuroscience and Physiology, Sahlgrenska Academy, University of Gothenburg, Box 455, 405 30 Göteborg, Sweden; 2 Sportrehab Sports Medicine Clinic, Göteborg, Sweden; 3 Department of Orthopaedics, Institution of Clinical Sciences, Sahlgrenska Academy, University of Gothenburg, Göteborg, Sweden; 4 Department of Orthopaedics, Sahlgrenska University Hospital, Mölndal, Sweden

**Keywords:** Anterior cruciate ligament, Reconstruction, Registry, Physical therapy modalities, Knee, Rehabilitation

## Abstract

**Purpose:**

To characterise patients who returned to knee-strenuous sports after an anterior cruciate ligament (ACL) reconstruction.

**Methods:**

Data from isotonic tests of muscle function and patient-reported outcome measures, Tegner activity scale (Tegner and Lysholm in Clin Orthop Relat Res 198:43–49, 1985), physical activity scale, knee injury and osteoarthritis scale and knee self-efficacy scale were extracted from a registry. The 157 included patients, 15–30 years of age, had undergone primary ACL reconstruction and were all involved in knee-strenuous sports, i.e. pre-injury Tegner of 6 or higher. Return to sport was studied in two different ways: return to pre-injury Tegner and return to knee-strenuous sport (Tegner 6).

**Results:**

Fifty-two patients (33 %), who returned to pre-injury Tegner, 10 months after surgery, were characterised by better subjective knee function measured with the knee injury and osteoarthritis outcome score (*p* < 0.05), compared with patients who did not. These patients also had higher perceived self-efficacy of knee function (*p* < 0.01), measured with knee self-efficacy scale. Eighty-four patients (54 %) who returned to knee-strenuous sports, i.e. Tegner 6 or higher, were characterised by higher goals for physical activity (*p* < 0.01) and higher self-efficacy of future knee function (*p* < 0.05). Strength measurements showed that women who returned to sports were stronger in leg extension than women who did not. No differences were found in Limb Symmetry Index for knee strength or jumping ability.

**Conclusion:**

Patients who returned to sports after ACL reconstruction had better subjective knee function and higher self-efficacy of knee function. Results highlight that further emphasis should be placed at psychological factors during rehabilitation of patients after ACLR.

**Level of evidence:**

II.

## Introduction

Far too many patients do not return to sports after an anterior cruciate ligament (ACL) rupture [9, 41, 44]. One potential long-term concern is that this can result in a too low sustainable lifelong physical activity. In a comprehensive systematic review, Ardern et al. [3] reported that 81 % of patients with an ACL reconstruction returned to some type of sport, while only 55 % returned to competitive sports participation. Further, more than 50 % of patients returning to a high level of competition reported that their performance was reduced compared with their pre-injury performance [15, 19, 26]. Consequently, it can be argued that these results could be an indicative of suboptimal treatment or a risk of future impairments and functional limitations for patients after an ACL reconstruction [10, 39, 40, 44]. There can be many reasons, interacting in a complex manner, why patients do not return to sports. Low self-efficacy beliefs, fear of re-injury and insufficient knee function are often discussed [26, 27, 41].

A return to physical activity or sports after ACL injury must be carried out safely, which puts pressure on the patient’s as well as responsible physician’s and physical therapist’s judgement [18, 39]. Safety can be defined as a minimal risk of a re-injury or a subsequent associated injury in the short term and with decreased risk of osteoarthritis in the long term. Return to sports is often seen as a main outcome when valuing a reconstruction or rehabilitation as successful [25]; however, not returning to sport per se should not be defined as unsuccessful. The literature has attempted to present guidelines with objective measurements to facilitate decision-making for the responsible physician and physical therapist about returning patients safely to sports in the short term and a sustainable physical activity in the long term [1, 21, 27, 39]. In spite of this, clinical difficulty still remains when assessing the time at which patients are ready to return to sports and at what level. In addition, there is an absence of clear criteria of progression in the rehabilitation literature, leaving the current practice of ACL rehabilitation inconsistent [17]. In order to try to find criteria for a safe, sustainable return to sports, different batteries of tests, consisting of various muscle function tests and patient-reported outcome measures (PROMs), have been used in the literature [14, 19, 25, 27, 28, 39]. Knowledge of treatment after ACL injury and reconstruction may be deemed to have increased, but more detailed characteristics are needed in relation to patients who return to sports and those who do not, respectively [39]. Population-based registry studies provide a unique source of information by containing large numbers of patients that are followed over a long period of time. The aim of this study was to utilise a rehabilitation outcome registry to characterise patients who returned to pre-injury knee-strenuous sports after ACL reconstruction. The hypothesis was that patients who return to knee-strenuous sports were characterised by better knee function, fewer knee-related symptoms and less impairment during daily activities, sports and recreation, as well as an enhanced quality of life and higher self-efficacy of knee function.

## Materials and methods

The study was performed as a prospective observational registry study based on data from an ACL rehabilitation outcome registry. The registry is based in the western part of Sweden. It was established in June 2009 and reports on rehabilitation outcomes for patients with an ACL injury and ACL reconstruction. The registry consists of two parts: a patient-reported section and a physiotherapist-reported section. Through a website, patients report demographic data and four validated PROMs: the Tegner activity scale [35], physical activity scale (PAS) [12], knee injury and osteoarthritis outcome score [32] and knee self-efficacy scale [36] to the database. The physiotherapist enters the results from tests of the patients’ muscle function. Predefined follow-ups are set at 10 weeks, 4, 8, 12, 18 and 24 months and then yearly up to 5 years, followed by every fifth year after ACL rupture or reconstruction. Participation in the rehabilitation outcome registry is voluntary for patients.

### Participants

**Fig. 1:**
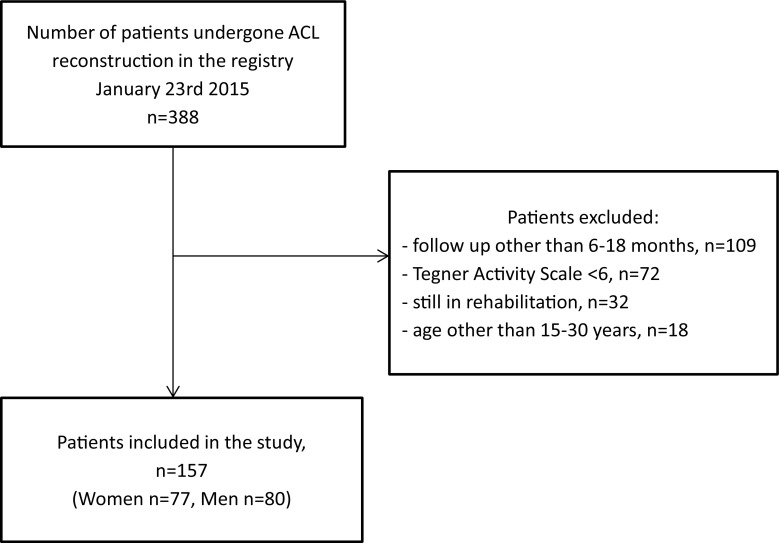
Flow chart of inclusion and exclusion criteria

Data were extracted from the rehabilitation registry. Patients with primary ACL reconstruction from 1 June 2009 to 23 January 2015 were eligible for inclusion (Fig. 1). Eligible patients had discontinued their rehabilitation 6–18 months after ACL reconstruction, and data from the follow-up closest in time to the patients’ discharge from the physiotherapy setting were used. Present definition of discontinued rehabilitation was based on clearance from responsible physiotherapist or the patient’s decision to discharge. A further inclusion criterion was a pre-injury self-reported physical activity level on the Tegner of 6 or higher, i.e. involvement in a knee-strenuous sport. Patients still undergoing rehabilitation were excluded as well as patients younger than 15 years or older than 30 years. Furthermore, the use of both the Tegner and PAS reinforced that the patients were regularly involved in sports [6, 39]. All the patients had completed a structured individualised rehabilitation programme at the same sports physiotherapy clinic.

### Procedure

#### Definition of return to physical activity

Return to physical activity was defined in two ways: one, patients who had returned to their pre-injury level of Tegner ± 1 [11, 22, 23] but a minimum of Tegner 6, and two, patients who had returned to a Tegner of 6 or higher, i.e. a knee-strenuous sport.

#### Muscle function

Evaluations of muscle function were performed with a battery of tests consisting of:

- Two reliable and valid isotonic tests for muscular strength, to reflect quadriceps and hamstring muscular power in knee extension and knee flexion [28]. The strength tests were performed in a knee extension and knee flexion weight training machine (Precor, Competition Line, Borås, Sweden). The average power was recorded through a linear encoder and calculated by Muscle Lab, a computerised muscle function measuring system (Ergotest Technology, Oslo, Norway). Tests were performed between 0º and 110° of knee flexion.
- Three reliable and valid single-leg tests for hop performance [14]: the vertical jump, the hop for distance and the side hop.

The results were presented as absolute values accounting for body weight and with the Limb Symmetry Index (LSI) [29]. In order for patients to perform the tests of muscle function, they had to be familiarised with the tests and have a current absence of pain from their knees during training. If criteria were not meet, the test leader made an assessment of the patient’s capability of performing the tests of muscle function.

#### Patient-reported outcome measures (PROMs)

Four validated PROMs: *Knee injury and osteoarthritis outcome score (KOOS)* [32], *knee self*-*efficacy scale (K*-*SES)* [36], *Tegner activity scale* [35], *physical activity scale (PAS)* [36] were used to evaluate factors that have been shown to be of importance for patients with an ACL injury [6, 9, 25]. Patients were asked to report their physical activity on Tegner and PAS for pre-injury, present and future goals.

Approval has been obtained from the Regional Ethical Review Board in Gothenburg, Sweden (registration number: 265-13). The study complies with the revised version of the Declaration of Helsinki [43]. Procedures are presented according to the STROBE Statement [42].

### Statistical analyses

Statistical analyses were performed using SPSS (version 22, 2013 SPSS Inc., Chicago, IL, USA). Descriptive statistics, reported as the mean, standard deviation and 95 % confidence intervals, were used for patient demographics and outcomes. An independent parametric, *t* test, and nonparametric tests, the Mann–Whitney *U* test, were used for between-group comparisons for demographic data, tests of muscle function and outcomes [20]. Alpha was set at *p* < 0.05.

## Results

### Return to pre-injury Tegner

Fifty-two of the 157 patients (33 %) reported that they had returned to their pre-injury Tegner ± 1 10 months on average after the ACL reconstruction. Group demographics and comparisons for women and men who had and had not returned to their pre-injury Tegner ± 1 are presented in Table 1.

**Table 1:** Demographics, comparisons and number of tests by gender for patients that had and had not returned to their pre-injury Tegner activity scale ± 1

Demographics	Women			Men		
	Returned (*n* = 23)	Not returned (*n* = 54)	*p* value	Returned (*n* = 29)	Not returned (*n* = 51)	*p* value
Months after surgery	9.9 ± 2.6	10.0 ± 3.5	n.s.	9.9 ± 3.3	10.6 ± 3.4	n.s.
Age						
Mean ± SD	20.8 ± 3.0	21.4 ± 3.8	n.s.	23.7 ± 4.5	23.3 ± 4.2	n.s.
Height						
Mean ± SD	172 ± 5.8	168 ± 5.3	0.004*	181 ± 7.6	181 ± 5.2	n.s.
Weight						
Mean ± SD	67 ± 8.1	62 ± 13.1	n.s.	77 ± 10.5	80 ± 9.2	n.s.
Pre-injury Tegner						
Median [range]	8 [6–10]	8 [6–10]	n.s.	9 [6–10]	9 [6–10]	n.s.
Mean ± SD	7.9 ± 2.3	8.2 ± 1.4		8.1 ± 1.9	8.8 ± 1.0	
Pre-injury PAS						
Median [range]	4 [3–4]	4 [2–4]	n.s.	4 [2–4]	4 [2–4]	n.s.
Mean ± SD	3.7 ± 0.5	3.6 ± 0.5		3.7 ± 0.5	3.8 ± 0.9	
Knee extension (*n*)	21	53		29	50	
Knee flexion (*n*)	21	53		29	49	
Vertical jump (*n*)	7	12		8	17	
Hop for distance (*n*)	14	25		18	23	
Side hop (*n*)	14	24		19	22	
KOOS (*n*)	23	54		27	51	

*Tegner* Tegner activity scale, *KOOS* knee injury and osteoarthritis outcome score, *PAS* physical activity scale

* Significant difference between groups, *p* < 0.05

**Fig. 2:**
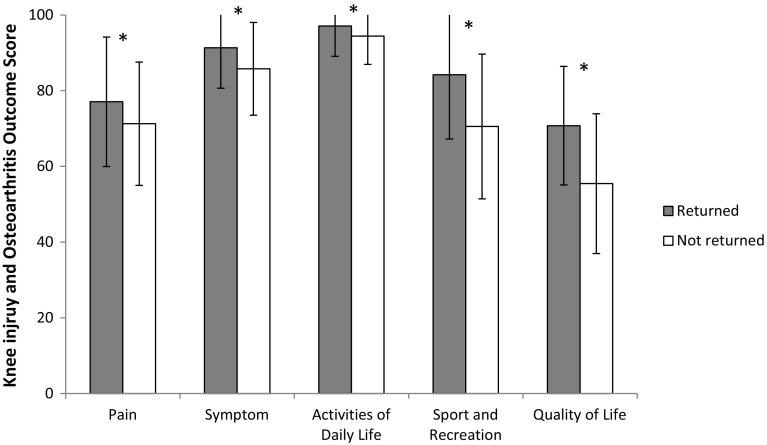
Knee injury and osteoarthritis outcome score subscale scores with SD for patients who had and had not returned to their pre-injury Tegner activity scale ± 1. *Significant difference between groups, *p* < 0.05

No significant difference in the LSI, with values between 90 and 97 %, was found for muscle function between patients who had returned and for patients who had not returned to their pre-injury Tegner ± 1. Subjective knee function as measured with KOOS differed significantly between groups for all sub-scales: pain (*p* = 0.038), symptoms (*p* < 0.001), ADL (*p* = 0.003), sport and recreation (*p* < 0.001) and quality of life (*p* < 0.001; Fig. 2). PROM scores stratified by returning to pre-injury Tegner ± 1 and gender are presented in Table 2.

**Table 2:** Patient-reported outcome measure scores by gender for patients that had and had not returned to their pre-injury Tegner activity scale ± 1

PROM Mean ± SD Median [range]	Women			Men		
	Returned (*n* = 23)	Not returned (*n* = 54)	*p* value	Returned (*n* = 29)	Not returned (*n* = 51)	*p* value
Tegner present	7.4 ± 2.3	4.4 ± 1.5	<0.001*	7.6 ± 1.9	5.1 ± 1.5	<0.001*
	8 [2–10]	4 [2–7]		8 [4–10]	4 [1–7]	
Tegner goal	8.1 ± 2.3	7.4 ± 2.4	n.s.	8.2 ± 2.5	8.8 ± 1.1	n.s.
	8 [2–10]	8 [5–10]		9 [4–10]	8 [5–10]	
PAS present	3.2 ± 1.1	2.7 ± 0.6	<0.001*	3.6 ± 1.3	2.6 ± 0.8	<0.001*
	3 [2–4]	3 [1–4]		3 [2–4]	3 [1–4]	
PAS goal	3.5 ± 1.1	3.3 ± 1.3	n.s.	3.8 ± 1.4	3.8 ± 0.4	n.s.
	4 [2–4]	4 [2–4]		4 [3, 4]	4 [2–4]	
K-SES present	6.9 ± 1.4	6.1 ± 1.4	0.005*	7.3 ± 0.8	6.5 ± 1.0	<0.001*
	9 [2–10]	8 [3–10]		9 [7–10]	7 [1–10]	
K-SES future	7.8 ± 1.9	6.8 ± 2.7	n.s.	8.0 ± 1.4	7.0 ± 1.9	0.026*
	9 [1–10]	8 [2–10]		8 [4–10]	7 [2–10]	
KOOS pain	80 ± 15	73 ± 16	<0.001*	77 ± 19	69 ± 14	<0.001*
	82 [43–100]	71 [36–100]		82 [32–100]	68 [43–100]	
KOOS symptoms	91 ± 13	86 ± 11	<0.001*	89 ± 11	84 ± 12	<0.001*
	92 [33–100]	89 [61–100]		94 [53–100]	86 [36–100]	
KOOS ADL	97 ± 10	94 ± 7	<0.001*	96 ± 9	94 ± 8	<0.001*
	100 [47–100]	97 [74–100]		99 [56–100]	97 [68–100]	
KOOS sport	80 ± 20	68 ± 22	<0.001*	83 ± 17	66 ± 21	<0.001*
	85 [10–100]	70 [15–100]		87 [50–100]	70 [10–100]	
KOOS QoL	68 ± 17	55 ± 18	<0.001*	72 ± 16	53 ± 20	<0.001*
	63 [13–94]	50 [19–100]		67 [50–100]	56 [13–100]	

*Tegner* Tegner activity scale, *PAS* physical activity scale, *K*-*SES* knee self-efficacy scale, *KOOS* knee injury and osteoarthritis outcome score

* Significant difference between groups, *p* < 0.05

Absolute values for the tests of muscle function and hop performance, accounting for body weight due to the difference seen in demographics, showed significantly better results for women who had returned, compared with women who had not returned to their pre-injury Tegner ± 1 for knee extension for injured (mean 3.2 W/kg; 95 % CI 2.7–3.5, respectively, mean 2.6 W/kg; 95 % CI 1.9–2.7, *p* = 0.010) and uninjured legs (mean 3.5 W/kg, 95 % CI 2.1–4.1, respectively, mean 2.9 W/kg, 95 % CI 2.3–3.1, *p* = 0.014) and side hop for injured (mean 0.7 hops/kg, 95 % CI 0.5–0.9, respectively, mean 0.5 hops/kg, 95 % CI 0.3–0.6, *p* = 0.012) and uninjured legs (mean 0.8 hops/kg, 95 % CI 0.5–0.9, respectively, mean 0.6 hops/kg, 95 % CI 0.4–0.6, *p* = 0.004). No differences in absolute values were seen between men who had and had not returned to their pre-injury Tegner ± 1 (Table 3).

**Table 3:** Absolute values for test of muscle function for injured and non-injured leg for men and women that had returned and not returned to pre-injury Tegner ± 1

Test of muscle function Mean ± SD	Women			Men		
	Returned	Not returned	*p* value	Returned	Not returned	*p* value
Knee extension IL (W/kg)	3.2 ± 1.0	2.6 ± 0.7	0.010*	4.1 ± 0.6	3.9 ± 1.0	n.s.
Knee extension NL (W/kg)	3.5 ± 0.9	2.9 ± 0.9	0.014*	4.4 ± 0.6	4.4 ± 0.9	n.s.
Knee flexion IL (W/kg)	1.9 ± 0.5	1.6 ± 0.4	n.s.	2.5 ± 0.5	2.4 ± 0.6	n.s.
Knee flexion NL (W/kg)	2.1 ± 0.5	1.8 ± 0.5	n.s.	2.9 ± 0.5	2.8 ± 0.4	n.s.
Vertical jump IL (cm/kg)	0.23 ± 0.04	0.21 ± 0.06	n.s.	0.26 ± 0.07	0.27 ± 0.07	n.s.
Vertical jump NL (cm/kg)	0.26 ± 0.04	0.27 ± 0.03	n.s.	0.29 ± 0.05	0.27 ± 0.06	n.s.
Hop for distance IL (cm/kg)	2.1 ± 0.4	1.9 ± 0.3	n.s.	2.0 ± 0.3	2.0 ± 0.3	n.s.
Hop for distance NL (cm/kg)	2.1 ± 0.3	2.0 ± 0.3	n.s.	2.1 ± 0.4	2.1 ± 0.4	n.s.
Side hop IL (n/kg)	0.7 ± 0.2	0.5 ± 0.2	0.012*	0.8 ± 0.1	0.7 ± 0.2	n.s.
Side hop NL (n/kg)	0.8 ± 0.2	0.6 ± 0.2	0.004*	0.8 ± 0.1	0.7 ± 0.1	n.s.

*IL* injured leg, *NL* non-injured leg

* Significant difference between groups, *p* < 0.05

### Return to Tegner 6 or higher

Of the 157 patients, 84 (54 %), 35 women and 49 men, returned to Tegner 6 or higher. Group demographics by gender are presented in Table 4. Women who had returned were significantly taller (+3.9 cm) and heavier (+6.6 kg) than women who had not returned (Table 4).

**Table 4:** Demographics, comparisons and numbers of tests for men and women who had and had not returned to Tegner activity scale 6 or higher, i.e. knee-strenuous sports

Demographics	Women			Men		
	Returned (*n* = 36)	Not returned (*n* = 41)	*p* value	Returned (*n* = 49)	Not returned (*n* = 31)	*p* value
Months after surgery						
Mean ± SD	10.2 ± 3.1	9.8 ± 3.4	n.s.	10.1 ± 3.1	10.8 ± 3.7	n.s.
Age						
Mean ± SD	21.6 ± 3.9	21.0 ± 3.2	n.s.	23.2 ± 4.0	23.8 ± 4.8	n.s.
Height						
Mean ± SD	170.9 ± 5.9	167.0 ± 5.1	0.007*	180.3 ± 5.8	183.1 ± 6.3	n.s.
Weight						
Mean ± SD	67.6 ± 8.4	61.0 ± 14.0	0.014*	77.6 ± 8.8	81.5 ± 10.9	n.s.
Pre-injury Tegner						
Median [range]	8 [6–10]	8 [6–10]	n.s.	9 [6–10]	8 [6–10]	n.s.
Mean ± SD	8.5 ± 1.1	8.0 ± 1.5		8.9 ± 1.0	8.1 ± 1.8	
Pre-injury PAS						
Median [range]	4 [3–4]	4 [2–4]	n.s.	4 [2–4]	4 [2–4]	n.s.
Mean ± SD	3.7 ± 0.5	3.7 ± 0.5		3.8 ± 0.4	3.7 ± 0.8	
Knee extension (*n*)	34	39		49	30	
Knee flexion (*n*)	34	39		49	29	
Vertical jump (*n*)	11	8		17	8	
Hop for distance (*n*)	20	19		29	12	
Side hop (*n*)	20	18		29	12	
KOOS (*n*)	35	41		47	31	

*Tegner* Tegner activity scale, *KOOS* knee injury and osteoarthritis outcome score, *PAS* physical activity scale

* Significant difference between groups, *p* < 0.05

**Fig. 3:**
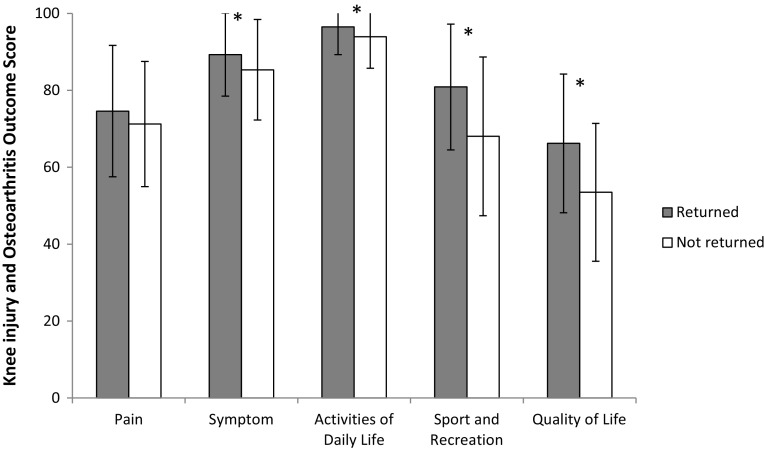
Knee osteoarthritis outcome score subscales with SD for patients that had and had not returned to Tegner activity scale 6 or higher, i.e. knee-strenuous sports. *Significant difference between groups, *p* < 0.05

No difference was found in the LSI, with values between 90 and 96 %, for the tests of muscle function between patients who had returned and patients who had not returned to knee-strenuous sports. Subjective knee function as measured with KOOS differed significantly between groups, where patients who had returned to knee-strenuous sports had a higher score for symptoms (*p* = 0.030), ADL (*p* = 0.017), for sport and recreation (*p* < 0.001) and for quality of life (*p* < 0.001), compared with patients who had not returned (Fig. 3). PROM scores stratified by returning to knee-strenuous sports and gender are presented in Table 5.

**Table 5:** Patient-reported outcome measures by gender for patients that had and had not returned to Tegner activity scale 6 or higher, i.e. knee-strenuous sports

PROM Mean ± SD Median [range]	Women			Men		
	Returned (*n* = 36)	Not returned (*n* = 41)	*p* value	Returned (*n* = 49)	Not returned (*n* = 31)	*p* value
Tegner present	7.3 ± 1.3	3.8 ± 1.0	<0.001*	7.4 ± 1.3	3.9 ± 0.9	<0.001*
	7 [6–10]	3 [2–5]		7 [6–10]	4 [1–5]	
Tegner goal	8.3 ± 1.9	7.2 ± 2.3	0.001*	8.9 ± 1.7	8.0 ± 1.7	0.006*
	8 [6–10]	7 [4–10]		9 [7–10]	7 [3–10]	
PAS present	3.2 ± 0.6	2.6 ± 0.6	<0.001*	3.1 ± 0.9	2.7 ± 1.4	0.002*
	3 [2–4]	2 [1–4]		3 [2–4]	2 [1–4]	
PAS goal	3.6 ± 0.9	3.3 ± 1.2	n.s.	3.8 ± 0.8	3.8 ± 1.1	n.s.
	4 [3, 4]	3 [3, 4]		4 [3, 4]	3 [2–4]	
K-SES present	7.0 ± 1.3	5.8 ± 1.3	<0.001*	6.9 ± 1.0	6.5 ± 1.0	n.s.
	9 [2–10]	6 [3–10]		8 [6–10]	7 [1–10]	
K-SES future	7.8 ± 2.3	6.5 ± 2.6	0.008*	7.8 ± 1.7	6.9 ± 2.0	0.048*
	8 [3–10]	6 [1–10]		8 [4–10]	6 [2–10]	
KOOS pain	75 ± 18	74 ± 14	0.030*	76 ± 17	70 ± 14	<0.001*
	79 [36–100]	71 [39–100]		79 [32–100]	68 [43–93]	
KOOS symptoms	91 ± 12	84 ± 11	<0.001*	88 ± 10	83 ± 13	<0.001*
	94 [33–100]	89 [61–100]		89 [53–100]	86 [36–100]	
KOOS ADL	97 ± 9	93 ± 7	<0.001*	96 ± 8	92 ± 9	<0.001*
	100 [47–100]	94 [74–100]		99 [56–100]	97 [68–100]	
KOOS sport	81 ± 18	62 ± 22	<0.001*	81 ± 15	62 ± 22	<0.001*
	85 [10–100]	60 [15–100]		85 [50–100]	70 [10–100]	
KOOS QoL	65 ± 19	52 ± 17	0.021*	68 ± 16	49 ± 19	<0.001*
	63 [13–100]	50 [19–81]		67 [38–100]	50 [13–88]	

*Tegner* Tegner activity scale, *PAS* physical activity scale, *K*-*SES* knee self-efficacy scale, *KOOS* knee injury and osteoarthritis outcome score

* Significant difference between groups, *p* < 0.05

The absolute values for the tests of muscle function and hop performance, accounting for body weight, show significantly better results for women returning to Tegner 6 or higher, i.e. knee-strenuous sports, for knee extension for injured (mean 3.0 W/kg, 95 % CI 2.3–3.2, respectively, mean 2.6 W/kg, 95 % CI 1.2–3.3, *p* = 0.032) and uninjured legs (mean 3.3 W/kg, 95 % CI 2.5–3.5, respectively, mean 2.8 W/kg, 95 % CI 1.7–3.6, *p* = 0.024) and knee flexion for non-injured legs (mean 2.1 W/kg, 95 % CI 1.8–2.3, respectively, mean 1.7 W/kg, 95 % CI 1.2–1.7, *p* = 0.017), compared with women who had not returned. The mean absolute values for men who had returned showed higher knee flexion strength in the injured leg (mean 2.5 W/kg, 95 % CI 2.2–2.8, respectively, mean 2.2 W/kg, 95 % CI 1.8–3.0, *p* = 0.039) compared with men who had not returned (Table 6).

**Table 6:** Mean absolute values ± SD and comparisons, accounted for body weight, of test for muscle function for men and women that had returned and not returned to Tegner 6 or higher, i.e. knee-strenuous sports physical activity

Test of muscle function Mean ± SD	Women			Men		
	Returned	Not returned	*p* value	Returned	Not returned	*p* value
Knee extension IL (W/kg)	3.0 ± 0.9	2.6 ± 0.7	0.032*	4.1 ± 0.8	3.8 ± 1.0	n.s.
Knee extension NL (W/kg)	3.3 ± 0.8	2.8 ± 0.6	0.024*	4.4 ± 0.7	4.3 ± 1.0	n.s.
Knee flexion IL (W/kg)	1.8 ± 0.5	1.6 ± 0.4	n.s.	2.5 ± 0.4	2.2 ± 0.7	0.039*
Knee flexion NL (W/kg)	2.1 ± 0.4	1.7 ± 0.5	0.017*	2.9 ± 0.4	2.8 ± 0.5	n.s.
Vertical jump IL (cm/kg)	0.22 ± 0.04	0.21 ± 0.07	n.s.	0.26 ± 0.06	0.28 ± 0.08	n.s.
Vertical jump NL (cm/kg)	0.26 ± 0.04	0.28 ± 0.01	n.s.	0.27 ± 0.06	0.29 ± 0.06	n.s.
Hop for distance IL (cm/kg)	2.0 ± 0.4	1.9 ± 0.3	n.s.	2.0 ± 0.3	2.1 ± 0.3	n.s.
Hop for distance NL (cm/kg)	2.1 ± 0.3	2.0 ± 0.3	n.s.	2.1 ± 0.3	2.1 ± 0.4	n.s.
Side hop IL (n/kg)	0.7 ± 0.2	0.6 ± 0.2	n.s.	0.8 ± 0.2	0.7 ± 0.2	n.s.
Side hop NL (n/kg)	0.7 ± 0.2	0.6 ± 0.2	n.s.	0.8 ± 0.1	0.8 ± 0.1	n.s.

*IL* injured leg, *NL* non-injured leg

* Significant difference between groups, *p* < 0.05

## Discussion

The main findings in this prospective observational registry study were that patients who returned to knee-strenuous sports had less impairment during daily activities, sport and recreation, enhanced knee-related quality of life and higher self-efficacy of knee function, at an average of 10 months of post-operative rehabilitation. Moreover, women who returned were stronger in terms of leg extension. The results partly confirm our hypothesis. Furthermore, the characteristics for returning to knee-strenuous sports found in the cohort will be presented in three groups: symptoms/impairment characteristics, muscle characteristics and psychological characteristics.

In this study, two different definitions of return to sports were used. The rationale for this was that *return to pre*-*injury Tegner* *±* *1* could exclude patients who actually do return successfully to a knee-strenuous sport. A broader definition of return, i.e. *return to Tegner 6 or higher*, was therefore chosen. For example, a patient with a pre-injury Tegner of 10 who *only* returned to a score of 6, 7 or 8 would be classified as non-successful using *return to pre*-*injury Tegner* *±* *1*, despite the fact that this patient had returned successfully to a knee-strenuous sport. The use of *return to Tegner 6 or higher* resulted in an increase in the return rate, which is in agreement with the literature [4]. Furthermore, patients who had not returned to a knee-strenuous sport had a lower future knee self-efficacy, potentially affecting patients’ motivation to reach a sufficient level of physical activity. Lower self-efficacy beliefs could, furthermore, partly explain the lower goal for future level of physical activity seen among patients who had not returned [38]. The use of *return to Tegner 6 or higher* also resulted in a reduction in numbers of significant differences in tests of muscle function between patients who had returned and patients who had not.

Symptoms/impairment factors, reflected by the KOOS, showed that patients who had returned reported that they had less pain, fewer symptoms and less impairment during activities of daily living, as well as less impairment during sport and recreation, compared with patients who had not returned. The finding of less pain could be considered interesting in regard to Heijne et al. [16], who showed that less pre-operative anterior knee pain was a predictor of better function in sports 12 months after ACL reconstruction. However, the results of fewer symptoms and less impairment would be considered to need further investigation, and whether these findings could be explained by less disabling injury to the patients’ knee or better performed rehabilitation.

The importance of psychological factors was illustrated by the fact that patients who had returned to knee-strenuous sports had higher self-efficacy beliefs and an enhanced knee-related quality of life, compared with patients who had not returned. These results are in line with Thomeé et al. [38], who found that knee self-efficacy was a predictor of a return to physical activity, symptoms and muscle function 1 year after ACL reconstruction. Patients who returned to knee-strenuous sports had approximately one step higher on knee self-efficacy, compared with patients who had not returned. One step has been recommended as a relevant difference between groups, even though a minimal clinically important difference has not been fully established [37]. It is therefore suggested that the results of the present study should be interpreted with some caution. Patients who had not returned showed lower self-efficacy beliefs, poorer knee-related quality of life, more impairment during sport and recreation and a lower future goal for sports participation compared with patients who had returned. These findings suggest that more emphasis should be placed on including psychological strengthening interventions during physiotherapy rehabilitation.

No major differences in muscle function, except for higher leg extension strength in returning women, were seen between patients who had returned to knee-strenuous sports and those who had not. This supports the current discussion that, in order to guarantee a successful return to sports, the recovery of muscle function alone is regarded as insufficient [39]. Regardless of the definition of return to sports used in this study, patients who had returned, as well as those who had not, had LSI values for muscle function around 90 %, which is usually regarded as sufficient [17, 39]. It is therefore suggested that, when evaluating muscle function, the physiotherapist should look beyond LSI values, which are commonly reported [8, 13]. For example, Eitzen et al. [7] found that a pre-operative asymmetry in quadriceps strength of more than 20 % predicted a poorer functional outcome 2 years after ACL surgery. In contrast, in the present study, women who had returned to knee-strenuous sport had higher knee extension strength compared with women who had not returned, despite the fact that no difference was seen between groups in terms of LSI values. In terms of muscle function, the importance of hop tests has previously been shown as a predictor of self-reported knee function 12 and 24 months after ACL reconstruction [24]. It is worth noting that, in the present study, no differences were seen between groups in terms of the hop tests. However, the number of patients who had performed the hop tests was low compared with the PROMs and strength tests. This may explain why no differences were seen between groups in terms of the hop tests.

In the present study, several factors that could influence the patient’s ability to return to sports are not evaluated. First, the present study has not evaluated compliance during rehabilitation. Risberg et al. [31] compared two different rehabilitation protocols after ACL reconstruction and suggested that high compliance with rehabilitation is important to the physiotherapy rehabilitation outcome.

Second, the possibility cannot be excluded that the differences in outcomes between patients who had returned and those who had not can be explained by surgical factors [5, 33].

Third, patients with associated injuries have been reported to have poorer knee function and more impairment in their injured knee [2, 34]. This implies that, in the present study, the differences seen between patients who returned and those who did not could be explained by the frequency and severity of associated injuries and their impact on the patients’ knee function and ability to return to sports. Oiestad et al. [30] found that, despite improvements in knee function outcome, patients with associated injuries had a significantly higher prevalence of osteoarthritis 10–15 years after ACL reconstruction. A standardised method for quantifying associated injuries is therefore warranted for future studies to enable the more specific sub-grouping of patients after ACL injury. This could also help physiotherapists and physicians to formulate more objective guidelines for determining when patients are ready for a return to physical activity.

The methodological limitations included the fact that no randomisation of patients or power calculation was performed. Rehabilitation programmes were individualised to suit the patients. Moreover, no blinding of patients, caregivers or assessors was used in the study. The caregiver and assessor for some patients could have been the same physiotherapist, and this may have influenced the patients’ performance positively or negatively. The loss of data from the follow-up of the hop test, due to patients being judged as unable to perform the tests, limits the opportunity to draw conclusions from the hop-test results.

Hypothetically, as a group, patients with an ACL injury may be too heterogeneous and thus limit the opportunity for studies to provide specific recommendations for physiotherapy rehabilitation or return to sports. In order to account for the heterogeneity, large studies are needed, in terms of the number of outcome measurements used, the number of patients included and the length of time for follow-up. Larger studies could provide more detailed information and generate more homogeneous sub-groups, e.g. in terms of gender, age, type and level of sports participation, associated injuries and compliance with rehabilitation. The use of a registry for rehabilitation variables, like that used in the present study, could thus be recommended for future studies.

## Conclusion

In this cohort, patients who returned to knee-strenuous sports on average 10 months after ACL reconstruction and physiotherapy rehabilitation were characterised as having fewer symptoms and less impairment during daily activities, sport and recreation, compared with those who had not returned to knee-strenuous sports. Patients who had returned also had a higher frequency and intensity of physical activity, higher knee self-efficacy and enhanced knee-related quality of life. Women who had returned to knee-strenuous sports were stronger in terms of leg extension than women who had not returned, despite both groups having LSI values above 90 %.
